# Exploring disorder correlations in superconducting systems: spectroscopic insights and matrix element effects

**DOI:** 10.3762/bjnano.15.19

**Published:** 2024-02-12

**Authors:** Vyacheslav D Neverov, Alexander E Lukyanov, Andrey V Krasavin, Alexei Vagov, Boris G Lvov, Mihail D Croitoru

**Affiliations:** 1 National Research Nuclear University MEPhI, Moscow 115409, Russian Federation; 2 National Research University Higher School of Economics, 101000 Moscow, Russian Federation; 3 Departamento de Física, Centro de Ciências Exatas e da Natureza,Universidade Federal de Pernambuco, Recife, Pernambuco, 50740-560, Brasil

**Keywords:** disorder, spatial correlations, superconductivity

## Abstract

Understanding the intricate interplay between disorder and superconductivity has become a key area of research in condensed matter physics, with profound implications for materials science. Recent studies have shown that spatial correlations of disorder potential can improve superconductivity, prompting a re-evaluation of some theoretical models. This paper explores the influence of disorder correlations on the fundamental properties of superconducting systems, going beyond the traditional assumption of spatially uncorrelated disorder. In particular, we investigate the influence of disorder correlations on key spectroscopic superconductor properties, including the density of states, as well as on the matrix elements of the superconducting coupling constant and their impact on the localization length. Our findings offer valuable insights into the role of disorder correlations in shaping the behavior of superconducting materials.

## Introduction

In contemporary quantum physics research, critical efforts revolve around understanding the fundamental principles governing correlations and their emergence. These correlations hold significant promise for the creation of a new class of materials endowed with highly desirable attributes. This pursuit is particularly pertinent within the field of highly correlated materials, exemplified by superconductors, where many factors, including disorder, can have profound effects. Although the interplay between disorder and such materials may seem extremely complex at first, the structural aspects of disorder can nevertheless serve as targets for manipulating the properties of superconducting materials [1–4].

Interest in the interplay between superconductivity and disorder has recently increased greatly due to new findings both in theory and in experiment [5–13]. Previous investigations primarily centered on probing the impact of disorder strength with a focus on improving superconducting characteristics. Consequently, it is now well established that increased levels of disorder can substantially improve key parameters of superconductivity. The studies have unveiled notable superconductivity enhancement in quasi-one-dimensional single crystals composed of weakly bound MoSe chains and sodium atoms [14]. Similarly, experiments with TaS_2_ monolayers have demonstrated an anomalous boost in superconductivity, which is also attributed to a presence of significant structural disorder [15]. Disorder-related effects have been implicated in the observed increase of the critical temperature in recently discovered NbSe_2_ superconducting monolayers [16]. Theoretical investigations attribute this enhancement to the disorder-induced multifractal structure of electronic wave functions [17–18], as revealed through numerical solutions of microscopic theory equations in low-dimensional samples [19–20].

In most materials, inhomogeneities exhibit a certain structure characterized by long-range spatial correlations. There is a growing recognition that such correlations exert profound alterations of the basic properties of disordered systems [21–41], including Bose–Einstein condensates [42–46] and superconductors [38,47–48]. For example, experimental work [47] has observed scale-invariant patterns in La_2_CuO_4+_*_y_* using a new kind of X-ray microscopy and has come to the conclusion that such fractal structures boost superconductivity. In particular, the work demonstrates that this material has a small number of very highly ordered regions and larger numbers of disordered regions, with a power-law distribution describing them. This is the hallmark of a scale-free distribution, which is typical of a fractal pattern where stripes with oxygen form a similar structure on all scales up to 400 μm.

The exploration of superconductivity in the presence of spatially correlated disorder has recently been initiated, as evidenced by the work of Neverov and co-workers [49]. This study has unveiled a significant influence of these correlations on the superconductive characteristics at the zero-temperature limit, where thermal energy represents the smallest energy scale within the investigated system. Specifically, the research has demonstrated that power-law correlations alter both the spatial distribution and the absolute value of the superconducting order parameter. Notably, an increase in the degree of correlation within the disorder potential is shown to augment superconductivity, aligning closely with experimental findings previously reported in [47].

This work focuses on a different aspect of the influence of the disorder correlations, investigating how the latter affect key spectral characteristics of a superconductor, that is, the energy level distribution. Recent scanning tunneling spectroscopy experiments on highly disordered amorphous superconductors [50] revealed that in the regime of superconductor–insulator transition the superconducting gap is stable, whereas the coherence peaks in the single-particle density of states (DOS) disappear. Following this observation, it was suggested that the system exhibits localization of Cooper pairs near the transition. In addition, in the presence of significant disorder, almost localized electronic wave functions show their fractal nature [50], which leads to an increase of the interaction matrix elements near the superconductor–insulator transition. This work elucidates how this mechanism, which controls superconductivity in strongly disordered materials, is modified when the disorder becomes correlated.

## Model

Superconductivity characteristics of a disordered superconductor are investigated using an attractive Hubbard lattice Hamiltonian with point interaction, introduced to model the *s*-wave Cooper pairing [51–52],


[1]

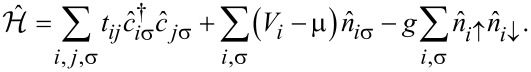



Here, the particle number operators 
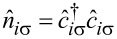
 at site *i* are expressed through the electron operators 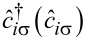 with spin σ. The tunneling amplitude *t_ij_* = −*t* is assumed nonzero only for the nearest sites, μ denotes the chemical potential, *V_i_* is the disorder potential, and *g >* 0 denotes the on-site superconducting coupling constant.

Because of common computational constraints, we consider a two-dimensional (2D) *N* × *N* square lattice with the 2D index *i* = (*i_x_*, *i_y_*) to denote lattice sites. Further, to maximally eliminate effects of the finite specimen dimensions, we assume periodic boundary conditions. The model is treated within the standard Hartree–Fock–Bogoliubov (HFB) mean-field approximation. Clearly, the mean-field results are not accurate for a strictly 2D system because of fluctuations. However, as our purpose is to describe a 3D system, where the fluctuations are not so crucially important, we assume that the mean-field results for the 2D model can serve as at least a qualitative guidance for 3D superconductors.

The mean-field HFB approximation for the model in Equation 1 gives rise to the Bogoliubov–de Gennes (BdG) equations which can be written as a matrix eigenvalue problem as [53–55]:


[2]

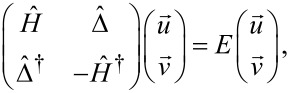



where *v_i_* = *v*(**r***_i_*) and *u_i_* = *u*(**r***_i_*) are components of the eigenvectors with **r***_i_* denoting the corresponding lattice point. Diagonal 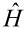 and off-diagonal 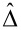 matrix operators have elements defined as [56–57]




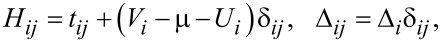




where δ*_ij_* is the Kronecker delta symbol. In this equation, the superconducting order parameter Δ*_i_* and the Hartree potential *U_i_* are determined through the self-consistency equations [6]:


[3]

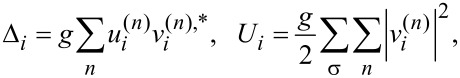



where the angular brackets ⟨…⟩ denote quantum mechanical averages, and the sum goes over the eigenfunctions of the BdG equations, labeled by index *n*. The Hartree self-consistency condition (Equation 3) modifies the effective potential that acts on electrons in the disordered system. This modification plays a pivotal role when dealing with strong disorder and cannot be disregarded, unlike in the case of weak disorder [5,58].

### Disorder model

To describe spatially correlated structural disorder in the system, we assume that in the inverse space the correlation form factor follows the power-law behavior in the limit of small *q*


[4]

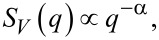



where the exponent α is called the degree of correlation. At α = 0, the disorder potential is completely uncorrelated (independent) at different points.

For efficient and controlled generation of Gaussian long-range power-law correlated disorder distribution, we use the Makse algorithm [59], which is essentially an improved version of the Fourier filtering method [22,60–62]. Although there are different approaches to implement this algorithm, we use one that can be summarized as follows. A particular disorder potential *V_i_* that satisfies the requirement set by Equation 4 is chosen using a model:


[5]

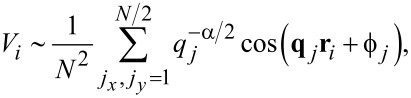



where **q***_j_* = (2π*j_x_*/*N*, 2π*j_y_*/*N*) is a discrete inverse space vector with *j_x,y_* = 1, …, *N* and *q_j_* = |**q***_j_*|, while ϕ*_j_* are random phases that are uniformly distributed in the interval [0,2π[. The strength of the disorder potential is characterized by its average amplitude, defined as


[6]

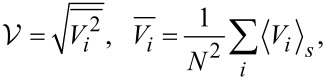



where the numerical averaging denoted by the overline is performed over both lattice sites and the statistical distribution. The brackets ⟨…⟩*_s_* indicate the statistical ensemble average. By construction, the resulting disorder correlation function has a power-law character. Moreover, it is Gaussian and stationary. Without loss of generality, we normalize the power-law series so that it has zero mean and the standard deviation as required by Equation 6.

### Numerical parameters

In the calculations, we take *N* = 50, which our calculations show is sufficiently large to mitigate finite-size effects. BdG equations (Equation 2) are solved together with the self-consistency conditions (Equation 3) in the usual way until the order parameter and the Hartree potential reach a predefined accuracy threshold [63–64]. Additionally, we determine the chemical potential μ from the condition that the average electron density *n*_e_ is fixed. In the calculations, we choose *n*_e_ = 0.875. This is quite far from the half-filling condition *n*_e_ = 1, at which a singular van Hove singularity appears in the DOS in the middle of the conduction band, which is an artefact of the 2D tight-binding model with only nearest neighbor direct hopping. This choice of *n*_e_ ensures that our results have general applicability beyond the constraints of the specific lattice configuration, making them equally valid for the 3D scenario. Actually, it is worth noting that the choice of the value of *n*_e_ is not important for our conclusions, as long as μ is far from the singularity [5–6].

The calculations are carried out for the strong coupling regime with *g* = 1*t*. The Debye energy is taken as ℏω_D_ = 5*t*, implying that the coupling is active for all single-particle states. We emphasize that in our study, all energy quantities are consistently represented in terms of the hopping integral *t*, while all lengths are standardized/expressed in units of the lattice parameter.

Lastly, the quantities of interest are statistically averaged over *N_s_* = 100 independent realizations of the disorder potential, characterized by selected specific values of 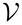 and α. (Note: The choice of *N* is solely dictated by the available computational resources; however, future studies should be aware of this limitation and may consider exploring a number of realizations beyond the specified number.) Details of the numerical procedure used to generate the disorder potential can be found in [65–66].

## Results and Discussion

We solve the problem for each realization of the random potential, obtained for three different values of the disorder correlation degree, namely α = 0 (uncorrelated potential), α = 1 (modest correlation with a finite correlation length), and α = 2 (strong correlation with the correlation length reaching the sample size). The solutions to the Bogoliubov–de Gennes (BdG) equations, encompassing an examination of their statistical attributes, spatial characteristics of the order parameter, and the superfluid stiffness, were elucidated in a prior study [49]. In the present study, we further explore the effects caused by correlated disorder and specifically elucidate the results related to the quasi-particle DOS, an aspect that has so far been overlooked in our earlier analysis. The quasi-particle DOS is determined by the following expression:


[7]

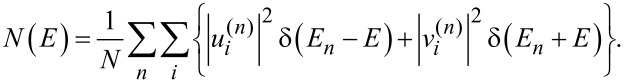



**Figure 1:**
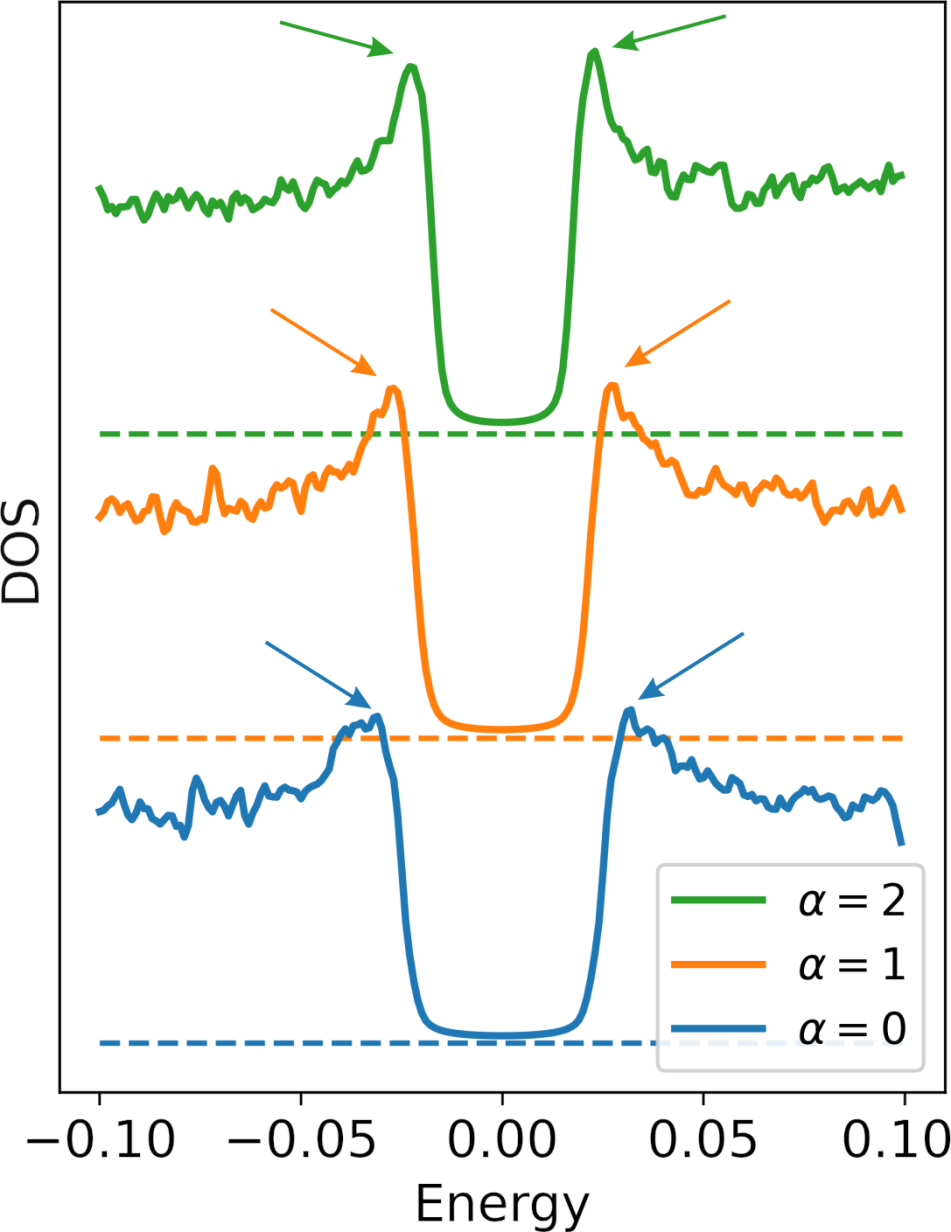
Density of states for different correlation strengths α and *V* = 1. The presence of correlations in disorder causes a noticeable reduction in the gap in the spectrum. Coherent peaks (indicated by arrows) appear in the density of states at the edge of the gap and show a dependence on α. The dashed lines specify the zero value for the corresponding DOS; the graphs are shifted vertically for clarity.

This quantity is ensemble-averaged over various realizations of the disorder potential, as illustrated in Figure 1 for three specific values of the correlation degree, α = 0, 1, and 2. The figure clearly indicates that both the gap and the two coherence peaks at its edges depend on α. Notably, the gap exhibits a discernible reduction with increasing α. In the case of uncorrelated disorder with α = 0, the gap is approximately 80% larger compared to the scenario with α = 2. Simultaneously, the coherence peaks gradually vanish with decreasing α, as demonstrated when comparing the results for α = 0 and α = 2. Furthermore, another distinct feature of the state behavior becomes apparent: In the context of uncorrelated disorder, the emergence of density of states into coherent peaks occurs at a slower rate. This gradual ascent aligns with the observations reported in [6], where states are pushed to higher energies because of the disorder. Considering the influence of the disorder correlation degree on coherent peaks within the quasiparticle density of states, it can be deduced that as α increases, the coherent peaks develop slightly higher. This is evident when comparing the outcomes for α = 0 and α = 2.

**Figure 2:**
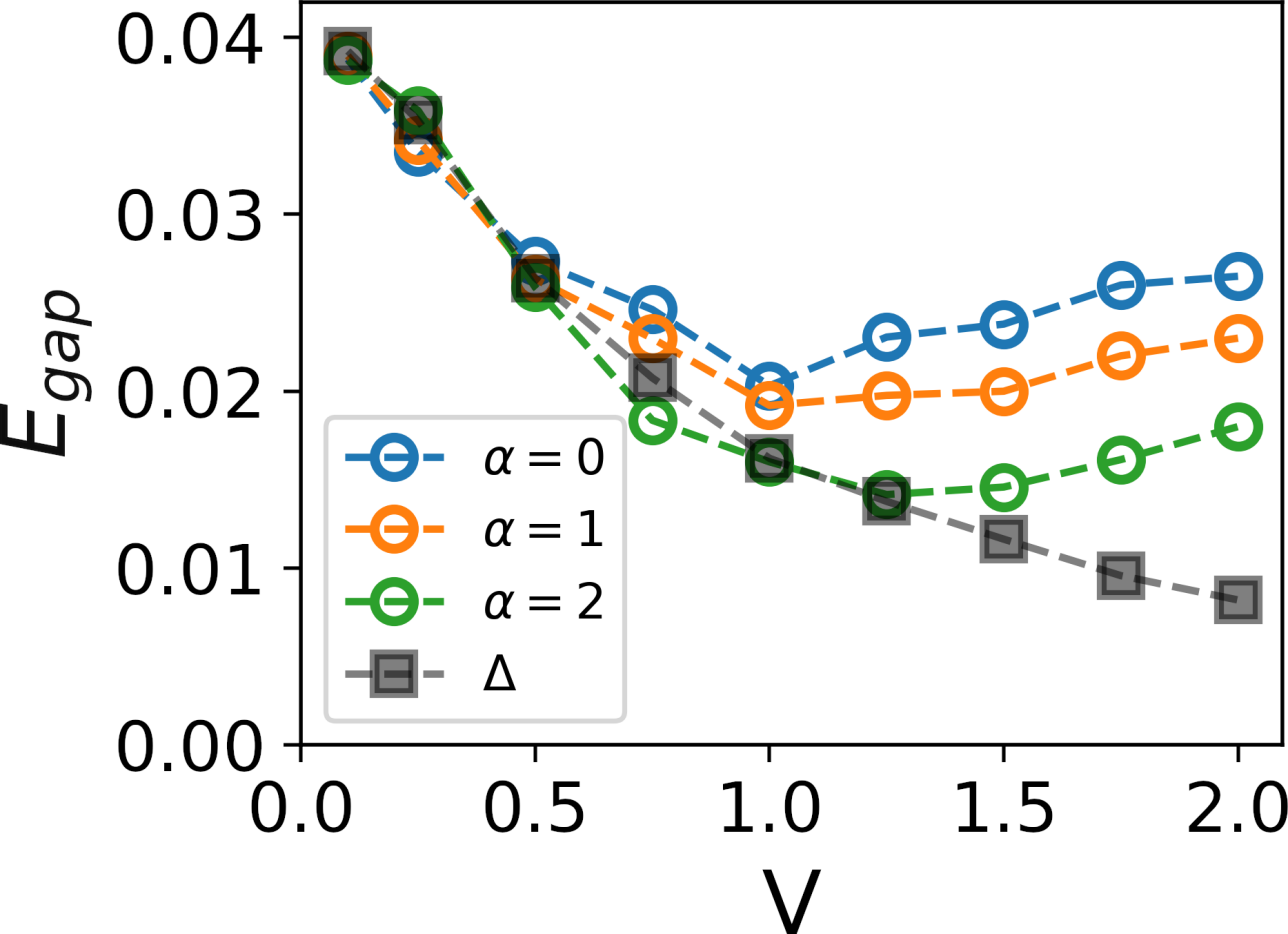
Dependence of the spectral gap on the disorder for various correlation strengths α. The evolution of the gap depends significantly on the level of correlations in the disorder. Notably, the gap’s dependence curve aligns closely with the dependence of the average value of local pairing Δ, which depends only slightly on the correlations of disorder.

To explain the reason for this behavior of the quasiparticle density of states, we present the spectral gap value as a function of the disorder strength in Figure 2. The energy gap is computed as the smallest eigenvalue of the BdG matrix in Equation 2. As anticipated, given the outcomes of [5], for uncorrelated disorder cases, the gap initially decreases, reaches a minimum, and then increases with strong disorder. The situation changes when considering a superconducting sample with correlations in disorder. It turns out that an increase in correlations in disorder leads first to a weakening and, with a further increase in α, to a complete suppression of the upward trend in strong disorder. In this figure, we also illustrate the behavior of the average order parameter as a function of the disorder strength (at α = 2). It is noticeable that at large α, the gap behavior tends to mirror that of the average order parameter.

**Figure 3:**
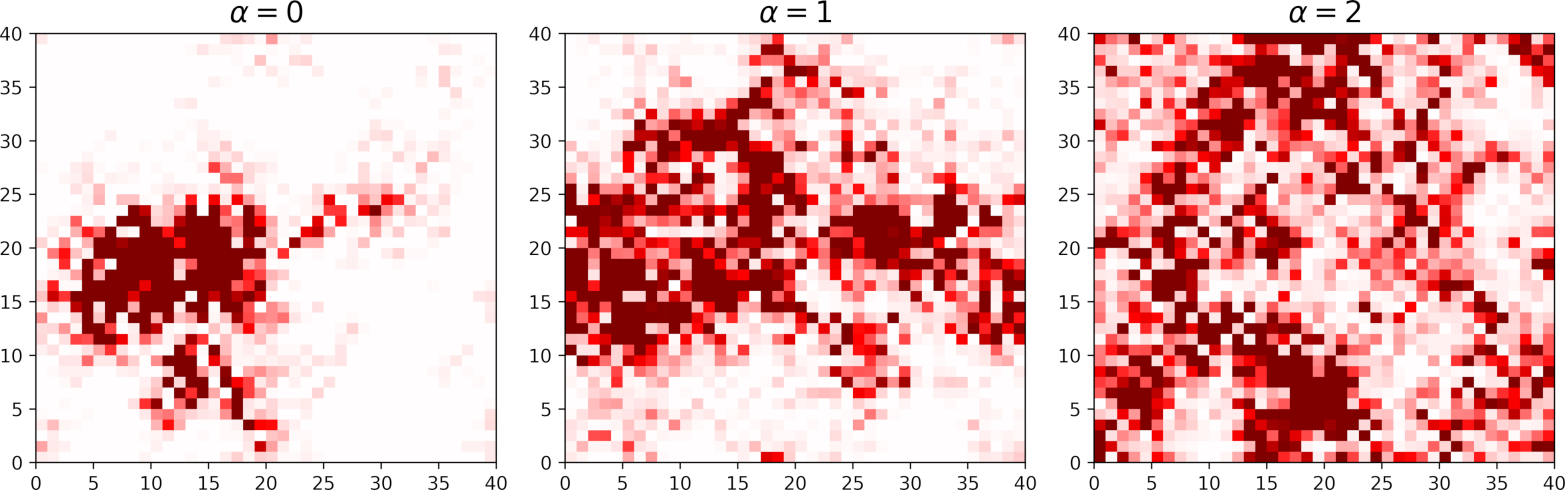
Lowest excitation state |*u*(*r_i_*)|^2^ + |*v*(*r_i_*)|^2^ for different correlation strengths α and *V* = 2. The spatial distribution map shows delocalization of the quasiparticle state with increasing correlations in disorder. The corresponding correlation lengths are ξ = 2.5, ξ_loc_ = 3.8 for α = 0, ξ = 3.0, ξ_loc_ = 4.4 for α = 1, and ξ = 4.0, ξ_loc_ = 6.2 for α = 2.

Given that the energy gap is determined by the smallest eigenvalue of the BdG matrix in Equation 2, it is of interest to visualize the wave function of that state. Figure 3 presents contour plots of the square of the quasiparticle wave function 
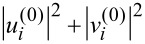
 for a specific disorder realization with a strength *V* = 2 and correlation degrees α = 0, 1, and 2. It becomes apparent that in the case of uncorrelated disorder, low-energy excitations form small islands, indicating their localization on superconducting islands (the same as in [6,67]). However, as α increases, percolation transitions start to manifest between these islands, eventually leading to a global superconducting transition with further increases in α, as previously discussed in [49]. This observation suggests that the enlargement of the spectral gap at high disorder strengths and low disorder correlation values is not linked to superconducting correlations; instead, it resembles a transition to an insulator state, thereby explaining the presence of a finite pairing gap at such disorder strength.

Understanding the process of percolation between superconducting grains with correlated disorder necessitates a comprehension of single-electron wave function behavior within a disorder potential. This analysis is rooted in the Anderson approximation solution of the BdG equations, wherein quasiparticle wave functions are proportional to single-particle wave functions within the same disorder potential. Further details of the Anderson approximation for granules and the corresponding investigation of interaction constant matrix elements are available in [68–69] and also in [6], where the method of matrix element analysis is referred to as the inverse participation ratio (IPR).

In cases of strong disorder, the coupling matrix elements


[8]

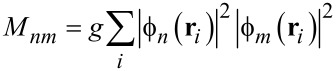



can be approximated as follows:


[9]

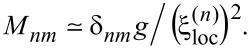



**Figure 4:**
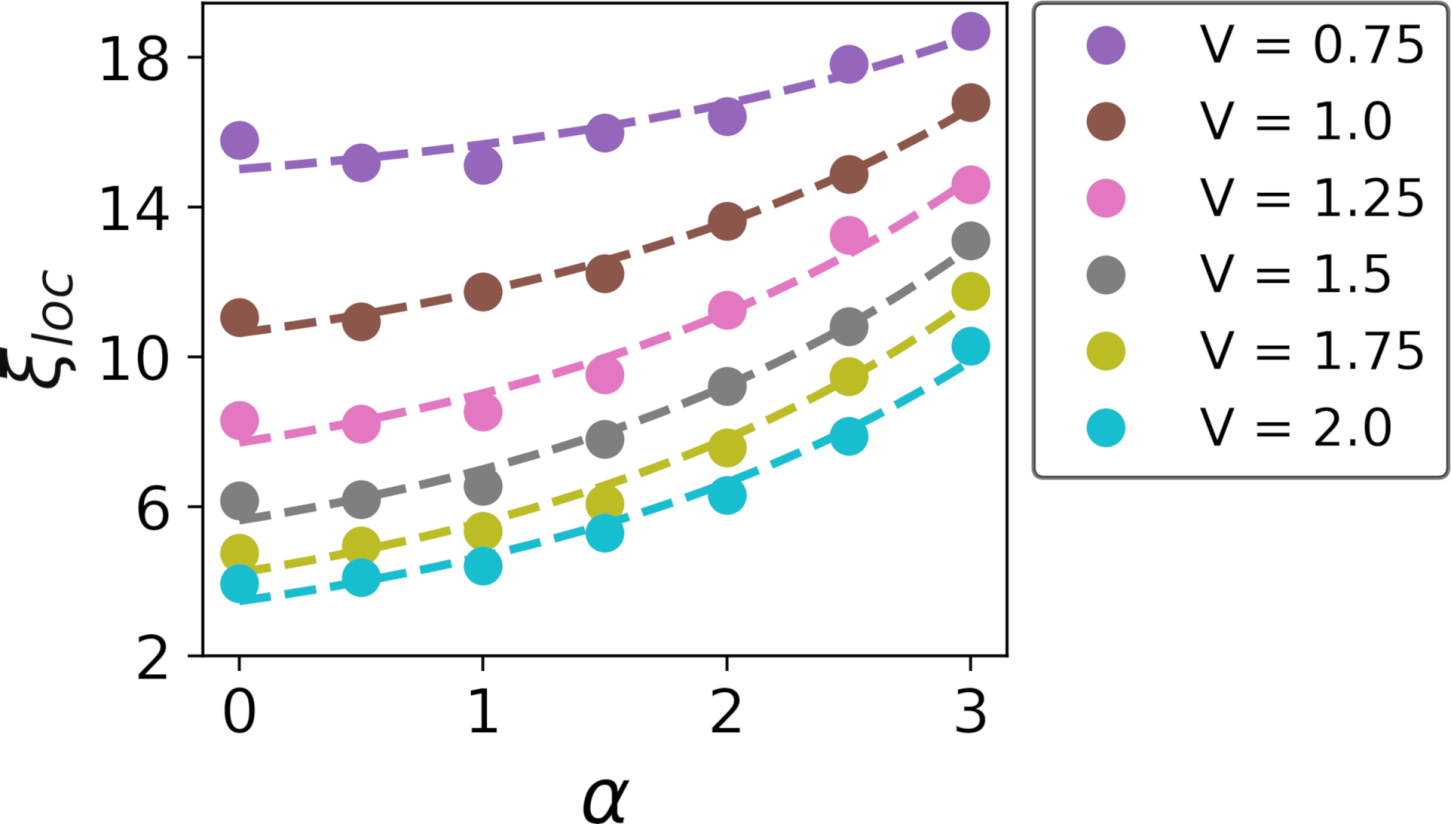
Localization length as a function of α (dots) and corresponding fits (dashed lines). Increasing the localization length preserves the ability of the system to remain in the superconducting state and to delay the moment of transition to an insulator state.

Here, 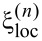 represents the localization length at the *n*-th level. As the primary contribution to pairing arises from time-reversal states near the Fermi level, Figure 4 illustrates the localization length 
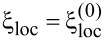
 as a function of the disorder correlation degree α. This figure clearly demonstrates a significant dependence of the localization length on both disorder strength and its correlations. As disorder strength increases, the length of correlations diminishes, whereas an increase in the degree of correlation enlarges the localization length.

The observed phenomenon of localization length plays a pivotal role in elucidating the spectral gap characteristics under conditions of substantial disorder. This relationship is particularly evident when considering the spectral gap behavior under significant disorder levels, as discussed in [6] for the uncorrelated disorder. While the spectral gap behavior under conditions of weak disorder depends primarily on the DOS at the Fermi level, its behavior under the influence of strong disorder is more accurately explained by taking also into account the concept of localization length. This explanatory framework remains applicable even in scenarios involving strong disorder with correlations.

It is worth emphasizing once again that at equivalent magnitudes of disorder strength, correlations exert a noticeable influence on the resulting physical picture. On the one hand, correlations cause changes in the behavior of superconducting characteristics similar to those arising from a decreased disorder; in particular, the transition to the disorder-induced spectroscopic gap (the transition to an insulator state) shifts toward larger disorder strength values as the correlation parameter α increases, effectively diminishing the disorder strength. On the other hand, the enhanced level of disorder correlations leads to a reduction in the spectroscopic gap, highlighting that an increase in the level of disorder correlations does not equate to a decrease in disorder.

## Conclusion

Our investigation on the impact of disorder correlations on superconductivity has yielded valuable insights into the behavior of the quasiparticle density of states within superconducting samples. The averaged quasiparticle density of states has revealed several important findings.

First, we observed a reduction in the spectral gap with an increase in the degree of disorder correlation. Regarding the impact of the disorder correlation degree on coherent peaks within the quasiparticle density of states, we noted that as α increased, the coherent peaks developed slightly higher, as demonstrated when comparing the results for α = 0 and α = 2. Regarding the disorder strength, the behavior of the gap was consistent with expectations based on previous research, with uncorrelated disorder cases initially leading to a decrease, reaching a minimum, and then increasing with strong disorder. However, when considering superconducting samples with correlations in disorder, an increase in correlations initially weakened and eventually suppressed the upward trend in strong disorder. This behavior was mirrored in the average order parameter.

The analysis of the behavior of coupling-constant matrix elements constructed on the single-electron wave function, rooted in the Anderson approximation solution of the BdG equations, provided further insights into the role of localization length in elucidating the spectral gap characteristics under conditions of substantial disorder. The localization length exhibited significant dependence on both disorder strength and its correlations. This relationship is crucial in explaining the spectral gap behavior, particularly at strongly correlated disorder levels.

In summary, our study highlights the intricate interplay between disorder correlations and the superconducting state characteristics. The observed behaviors in the quasiparticle density of states, spectral gap, and localization length contribute to our understanding of superconductivity in disordered systems and pave the way for further investigations in this intriguing field of condensed matter physics.

## Data Availability

The data that supports the findings of this study is available from the corresponding author upon reasonable request.
